# Assessing the Yield of Reduced Duration-EEG Recordings in Children: A Cross-Sectional Study

**DOI:** 10.7759/cureus.64233

**Published:** 2024-07-10

**Authors:** Nikita Agarwal, Rakesh Jain

**Affiliations:** 1 Pediatrics, All India Institute of Medical Sciences, Raipur, Raipur, IND; 2 Pediatric Neurology, Fortis Hospital, New Delhi, IND

**Keywords:** electroencephalography (eeg), encephalopathy, epilepsy, eeg duration, eeg in children

## Abstract

Introduction

An EEG is an important tool in the diagnosis of neurological diseases. Performing an EEG on children can be challenging due to their tendency to not cooperate for the recommended duration. We aim to optimize the duration of EEG recording in children by finding the optimal duration of recording.

Materials and methods

A single-center prospective observational study was done after appropriate ethical clearance. Children aged 0-14 were recruited and examined, and the recommended EEG was done. Data were collected and analyzed.

Results

Of the 112 EEGs analyzed, 29 EEGs were normal, i.e., no diagnostic anomaly was noticed. In the remaining 83 EEGs, if the duration of the EEG was reduced to 20 minutes, it resulted in missing the diagnostic anomaly in 20 cases (24.1%; 95% CI: 11.2%-26.2%).

Reducing the duration of the EEG recording to 10 minutes resulted in missing 63 of the diagnostic anomalies (75.9%; 95% CI: 46.6%-65.6%). Of the 86 drug-induced EEGs, 22 were normal (25.6%; 95% CI: 16.8%-36.1%). Of the 24 routine EEGs, seven were normal (29.2%; 95% CI: 12.6%-51.1%). Of the two sleep-deprived EEGs, neither was normal (0.0%; 95% CI: 0.0%-84.1%).

Conclusion

In our study, we observed that optimization of the duration of EEG recording can be done to 20 minutes in all populations. We also observed that if we find a diagnostic abnormality early during EEG recording, then continuation of the EEG may not be necessary to make a valid report. Having said so, having a negative EEG may not necessarily rule out a diagnosis. We did not find the superiority of any of the EEG protocols over others, as their yield was comparable.

## Introduction

An EEG is a crucial diagnostic tool for patients experiencing unexplained loss of consciousness, seizure activity, or neuro-psychiatric phenomena. It detects brain waveforms and patterns based on ion interactions and neuron-firing activities. The most common indication for EEG is unprovoked seizures, which have an annual incidence of 23-61 per 1,000 person-years [1].

Untreated seizures can lead to learning disabilities, behavioral disorders, depressive personalities, and a drop in the health-related quality of life index [2-3]. An EEG is essential for diagnosing and localizing lesions in viral encephalitis and space-occupying lesions, but not all interictal discharges on the EEG are indications for treatment. An EEG recording can be diagnostic and helpful in localizing the disease if the discharges are produced from a specific part of the brain. In pediatric patients and those with a generalized epileptiform presentation, EEG abnormalities are more frequently picked up [4]. The American Clinical Neurophysiology Society (ACNS) recommends a minimum of 20 minutes of EEG recording, but the optimum duration is not established. Most interictal EEG recordings give a waveform suggestive of diagnosis within the first 24 hours, with 37% picked up in the first 20 minutes. Factors affecting EEG yield include sleep deprivation and drug-induced EEG recording. Misdiagnosis and overdiagnosis can lead to unnecessary treatment and side effects of anti-seizure medication. A study found that extending the duration of routine EEG recording by 20 minutes only increased the yield by 11% in subjects older than 14 years of age [5].

The International League Against Epilepsy (ILAE) recommends a minimum recording of 30 minutes in pediatric subjects. Recurrent observation of interictal EEG abnormalities suggests a more severe disorder and should be treated more vigilantly. Factors affecting EEG yield include sleep deprivation and drug-induced recording. There is no single protocol recommended for optimal EEG recording duration in children, aiming to reduce costs and improve diagnostic tool efficiency [6].

An EEG is a crucial tool for investigating epilepsy, documentation, and treatment. However, there is no set protocol for recording duration in Indian children to minimize false positives and false negatives. Studies show that extending EEG recording duration may lead to interictal spikes, but it's difficult to determine its significance [7]. A 30-minute EEG may be distorted if the child wakes up before 30 minutes, causing distress and resource mismanagement. The goal of this study is to optimize recording duration while maximizing yield with minimal investment.

The primary objective of this study is the assessment of the yield of a reduced duration of EEG (from 30 minutes to 20 minutes and 10 minutes), and the secondary objective is the comparison of the yield of different EEG protocols.

## Materials and methods

**Figure 1:**
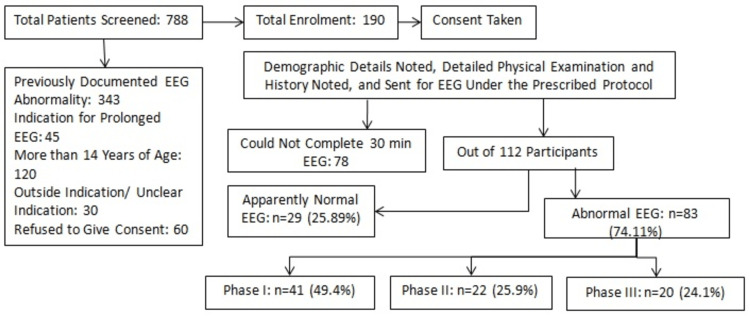
A diagram outlining the study flow

This observational study was carried out at the pediatrics department of Fortis Memorial Research Institute, Gurgaon, India, from February 2018 to December 2019. Previous research indicated that reducing the duration of the EEG led to a 6.42% increase in missed diagnoses. By using the formula n = (Z^2^ x P(1 - P))/e^2^, with Z = 1.96 as the standard deviation value for two standard deviations for a 95% confidence level, P representing the expected true proportion, and e as the desired precision, a sample size of 87 was calculated. However, a sample size of 100 was chosen for the study. The study flow is depicted in Figure 1.

The study included children under 14 years old who were referred for an EEG by a neuro-physician at the center. Children with prior EEG abnormalities or those requiring prolonged EEG monitoring (overnight and/or 24 hours) were excluded.

Ethical approval was granted by the institutional ethical committee (IEC) of Fortis Memorial Research Institute, Gurgaon, India (IEC code no.: 2018-008TH-22). Informed consent was obtained from the parents or guardians of all participating children, while assent was obtained from children over eight years old.

Patients and children meeting the inclusion criteria and not meeting the exclusion criteria at the Department of Paediatric Neurology outpatient department (OPD)/in-patient department (IPD), Fortis Memorial Research Institute, Gurgaon, India, were included in the study after providing informed and written consent. Patient care was maintained throughout the study. Upon enrollment, a thorough history was taken, including presenting complaints, medical history, birth history, and developmental history. A detailed central nervous system (CNS) examination and anthropometry were conducted. A child's EEG was recorded for 30 minutes during wakefulness, natural sleep, or drug-induced sleep, with readings taken at different intervals. Any abnormalities were documented.

Data were collected on specific forms and entered into a study binder. Categorical variables were presented as numbers and percentages, while continuous variables were presented as mean ± standard deviation (SD) and median. Data normality was assessed using the Kolmogorov-Smirnov test, with non-parametric tests used for non-normal data. Data were coded, entered, and analyzed using SPSS Statistics for Windows, Version 17.0. (SPSS Inc., Chicago, IL). The Student's t-test was used to compare mean values between intervention and control groups for continuous variables, while the chi-square test was used for variables like age and sex. A p-value of <0.05 was considered significant. It is important to clarify that patients and the general public were not involved in the study's design, data collection, or analysis.

## Results

In the study period, 788 children were screened, and 112 were eventually included after exclusions. Among the included children, the majority (44.6%) belonged to the one- to five-year-old age group; 58% of them were biological males. The median weight was 15 kg (interquartile range (IQR): 16.75 kg) and the median height was 93 cm (IQR: 31.75 cm). Out of the 112 subjects, seven (6.3%) had macrocephaly, and 24 (21.4%) had microcephaly. Regarding additional assessments, three (2.7%) children underwent EEG due to abnormal behavior; 87 (77.7%) had seizures or seizure-like activity; 12 (10.7%) had focal CNS abnormalities; and 10 (8.9%) had other EEG indications. Sixty-eight (60.7%) subjects had no relevant history. Developmental delays were observed in various domains among the children, with 27 (24.1%) diagnosed with global developmental delays. Details of CNS examination findings are presented in Table 1, where 70 (62.5%) children had abnormalities, while 42 (37.5%) had no focal neurological deficits.

**Table 1:** Details of the central nervous system examination of the included children

Abnormal findings	Frequency	%
Cranial nerve paralysis/palsy (single/ multiple)	17	15.2%
Tone abnormality (in at least one limb)	39	34.8%
Reduced power (in at least one limb)	33	29.5%
Absent superficial reflexes	10	8.9%
Delayed deep tendon reflexes (DTRs)	16	14.3%
Exaggerated DTRs	33	29.5%
Muscle wasting	10	8.9%
Abnormal cerebellar examination	27	24.1%

Among the 112 EEGs that were examined, 29 were found to be normal, indicating the absence of any diagnostic anomalies. In the remaining 83 EEGs, shortening the duration of the recording to 20 minutes led to the oversight of diagnostic anomalies in 20 cases, accounting for 24.1% (95% confidence interval (CI): 11.2%-26.2%).

Of the 112 EEGs, 69 (61.6%) helped reach the diagnosis in the subject, while 43 (38.4%) required repeat EEG and/or alternate protocol EEG to aid in diagnosis. Details of each protocol's diagnostic yields are summarized in Table 2.

**Table 2:** Proportion of diagnostic yields of different EEG protocols

Protocol	Frequency of diagnostic EEG	%
Sleep-deprived EEG	1	50.0%
Drug-induced EEG	53	61.6%
Routine EEG	15	62.5%

When the duration of the EEG recording was further reduced to 10 minutes, a total of 63 diagnostic anomalies were missed, representing 75.9% (95% CI: 46.6%-65.6%). Out of the 86 drug-induced EEGs (drug-induced means that a child was induced to sleep using an age- and weight-appropriate triclofos oral dose, and then the EEG was recorded), 22 were classified as normal, making up 25.6% (95% CI: 16.8%-36.1%). As for the 24 routine EEGs, seven were deemed normal, with a percentage of 29.2% (95% CI: 12.6%-51.1%). Lastly, the two sleep-deprived EEGs did not show any normal results, accounting for 0.0% (95% CI: 0.0%-84.1%).

The details of the effect of shortening EEG duration on various indications are summarized in Table 3.

**Table 3:** Effect of shortening the duration of EEG recording on various diagnoses

Category of diagnosis	Missed on shortening EEG duration to 10 minutes	95% confidence interval (CI)	Missed on shortening EEG duration to 20 minutes	95% CI
Seizure disorder	14 (41.2%)	26.6%-60.4%	6 (18.8%)	5.2%-32.3%
Status epilepticus/encephalopathy	0 (0%)	-	0 (0%)	-
Post-ictal	5 (83.3%)	53.1%-100%	2 (33.3%)	0%-71.7%
Infarction/space-occupying lesion	8 (53.3%)	28.1%-78.6%	2 (13.3%)	0%-30.5%
Syndromic	1 (6.7%)	0%-19.5%	1 (6.7%)	0%-19.5%
Undetermined	4 (80%)	44.9%-100%	1 (20%)	0%-55.6%

## Discussion

In 2013, Badry [8] conducted a study and discovered that within the first 20 minutes, 45% of the participants showed diagnostic abnormalities. This percentage increased by 10% in the following 40 minutes. Therefore, a total of 55% of abnormalities were detected within the first hour. Theither et al. [9] also conducted a study and found that 90% of interictal epileptiform discharges (IEDs) were recorded within 18.5 minutes of EEG recording, regardless of whether it was a standard or sleep-deprived EEG. Reardon et al. [10] conducted a study where they observed that reducing the duration of EEG from 25 to 15 minutes resulted in a 6.24% decrease in missed diagnoses. If we compare these data with our study, reducing the duration of EEG to 20 minutes resulted in missing the diagnostic anomaly in 20 cases (p< 0.05). Furthermore, reducing the duration to 10 minutes resulted in missing 63 diagnostic anomalies (p< 0.05). In the study by Miskin et al. [11], out of the 40-minute EEGs, 37 were abnormal, and 89% of these abnormalities were identified within the first 20 minutes. However, our results do not align with these data.

Losey and Uber-Zak [12] conducted a study that supported the findings of our current study. They found that reducing the duration of the EEG from 40 minutes to 20 minutes resulted in missing 47% of abnormal EEGs. On the other hand, Faulkner et al. [13] focused on the latency of the first abnormal diagnostic wave (IEDs), and their data cannot be directly compared to our study. They found that 48% of diagnostic abnormalities were detected within the first four hours, and this percentage only increased to 58% in the next four hours. It is important to note that Faulkner et al. [13] only included patients who required IED recording. Lastly, Burkholder et al. [14] found that only 4.8% of their EEGs showed positive results beyond 60 minutes. Their findings were similar to those of Reardon et al. [10] in 1999.

In the study conducted by Craciun et al. [15], similar to the work of Reardon et al. [10], the focus was on determining the percentage of abnormal waves missed on the EEG when the duration of the EEG was shortened from 30 minutes to 20 minutes for routine EEGs and from 60 minutes to 30 minutes for sleep EEGs. Their findings indicated that the minimum duration required for a routine EEG is 20 minutes, while for a sleep EEG, it is 60 minutes. However, they did not provide specific percentages in their data analysis. In our current study, within the category of status epilepticus/encephalopathy, all cases showed abnormalities in Phase I. In the syndromic disorders category, five cases (83.3%) tested positive in Phase I, none were positive in Phase II, and one case (16.7%) was positive in Phase III. These results align with the findings of Doudoux et al. [16] from 2017, where a higher percentage of positive results were observed in individuals with epilepsy syndromes compared to spells and focal cases. The number of diagnostic abnormal EEGs decreased from 121 (70.8%) in period 1 to 2 (1.7%) in period 5 as the study progressed. Our study results are consistent with those of Craciun et al. [15], Losey and Uber-Zak [12], Doudoux et al. [16], Miskin et al. [11], Reardon et al. [10], and Theither et al. [9].

The study's strengths lie in the diverse population of children selected, distinguishing it from previous studies that included a mix of childhood and adult EEG recordings. Conducted in Indian settings, where data is scarce, this study rigorously screened a significant number of children to ensure those with no prior EEG abnormalities were included, minimizing bias.

The study's constraints encompass the possibility of a more extensive sample size to further investigate particular facets of EEG, like pinpointing profile-specific triggers. Additionally, the population had some foreign children due to the institute's focus on medical tourism; therefore, an all-Indian group was not examined. Subsequent research may contemplate broadening the scope of the study to encompass the 45 children requiring extended EEG monitoring, thus yielding a more comprehensive dataset.

In our investigation, we suggest extending the EEG duration if no abnormal waves are detected in cases with high suspicion while excluding other potential diagnoses. Our rate of positive results is around 74% in a 30-minute EEG. We speculate that the rate could have been higher if we had conducted EEG recordings for one hour or longer. Therefore, a normal 30-minute EEG does not definitively rule out epilepsy, especially in cases with strong clinical suspicion. Additional provocative measures, such as drug-induced EEG and sleep-deprived EEG, should be considered.

## Conclusions

In the course of our investigation, we observed that it is feasible to streamline the duration of EEG recording to 10 minutes for certain demographics, such as children with non-convulsive status epilepticus and encephalopathy. Having said this, it is not advisable to concur with a negative report if the duration is reduced to less than 20 minutes in most diagnoses, such as unprovoked seizures. Additionally, we found no discernible advantage of one EEG protocol over another, as their efficacy was comparable. This may be explored further with more dynamic study designs.
